# Temperature as a risk factor of emergency department visits for acute kidney injury: a case-crossover study in Seoul, South Korea

**DOI:** 10.1186/s12940-019-0491-5

**Published:** 2019-06-14

**Authors:** Satbyul Estella Kim, Hyewon Lee, Jayeun Kim, Young Kyu Lee, Minjin Kang, Yasuaki Hijioka, Ho Kim

**Affiliations:** 1 Center for Climate Change Adaptation, National Institute for Environmental Studies, 16-2 Onogawa, Tsukuba, 305-8506 Japan; 2 Institute of Health and Environment, Seoul National University, 1 Gwanak-ro, Gwanak-gu, Seoul, 08826 Republic of Korea; 3 Division of Nephrology, Department of Internal Medicine, National Health Insurance Service Ilsan Hospital, Goyang, Republic of Korea; 4 Research and Analysis Team, National Health Insurance Service Ilsan Hospital, Goyang, Republic of Korea; 5 Department of Health Sciences, Graduate School of Public Health, Seoul National University, 1 Gwanak-ro, Gwanak-gu, Seoul, 08826 Republic of Korea

**Keywords:** Ambient temperature, Acute kidney injury, Emergency department visit, Case-crossover design, Exposure-response curve

## Abstract

**Background:**

Previous studies show that escalations in ambient temperature are among the risk factors for acute kidney injury (AKI). However, it has not been adequately studied in our location, Seoul, South Korea. In this study, we aimed to examine the association between ambient temperatures and AKI morbidity using emergency department (ED) visit data.

**Methods:**

We obtained data on ED visits from the National Emergency Medical Center for 21,656 reported cases of AKI from 2010 to 2014. Time-stratified case-crossover design analysis based on conditional logistic regression was used to analyze short-term effects of ambient temperature on AKI after controlling for relevant covariates. The shape of the exposure–response curve, effect modification by individual demographic characteristics, season, and comorbidities, as well as lag effects, were investigated.

**Results:**

The odds ratio (OR) per 1 °C increase at lag 0 was 1.0087 (95% confidence interval [CI]: 1.0041–1.0134). Risks were higher during the warm season (OR = 1.0149; 95% CI: 1.0065–1.0234) than during the cool season (OR = 1.0059; 95% CI: 1.0003–1.0116) and even higher above 22.3 °C (OR = 1.0235; 95% CI: 1.0230–1.0239).

**Conclusions:**

This study provides evidence that ED visits for AKI were associated with ambient temperature. Early detection and treatment of patients at risk is important in both clinical and economic concerns related to AKI.

**Electronic supplementary material:**

The online version of this article (10.1186/s12940-019-0491-5) contains supplementary material, which is available to authorized users.

## Background

Increasing recognition of climate change and global warming has led to a growing interest of researchers in assessing the potential mechanisms by which it may influence health [1]. The adverse impacts of ambient temperature on health have been described in numerous epidemiological studies [2–4], and some studies have reported that escalations in temperature or heat (waves) are among the risk factors for acute kidney injury (AKI) [5–10].

**Fig. 1:**
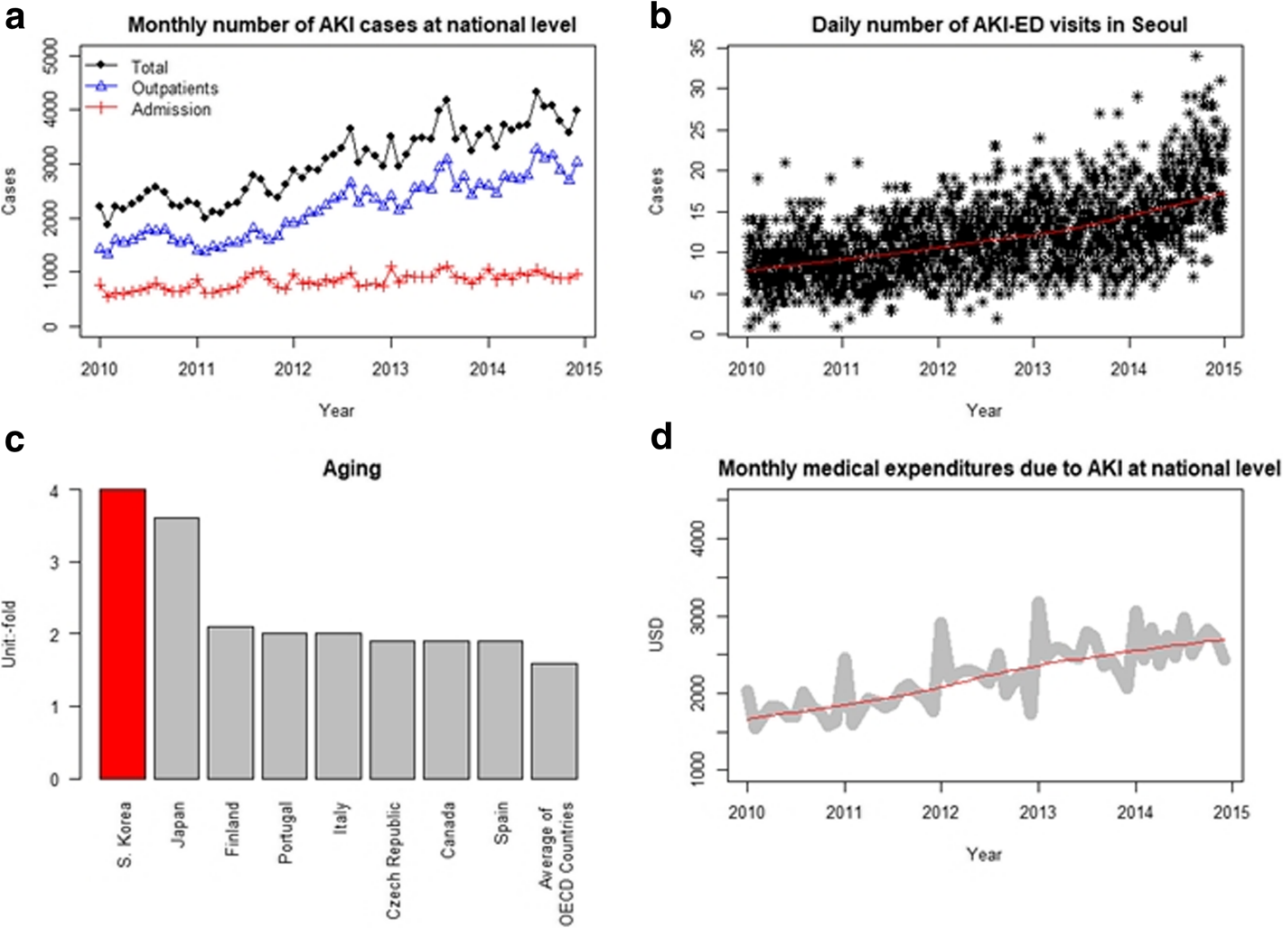
Prevalence of acute kidney injury (AKI) in South Korea. **a** Time trend of AKI at the national level in South Korea. **b** Daily number of AKI-emergency department (ED) visits in Seoul; analyzed data set. **c** Rate of ageing individuals among Organization for Economic Co-operation and Development (OECD) countries above the average. Proportion of the elderly population (age > 65 years) in 2013 when set at 1970 to 1. Data were obtained from the Institute for Industrial Economics and Trade of Korea. **d** Medical expenditures due to AKI at national level. Data were obtained from the Health Insurance Review and Assessment Service of Korea

AKI is defined as “a sudden episode of kidney failure or kidney damage that happens within a few hours or a few days” regardless of the cause [11, 12]. AKI is a complex and serious health condition, and its occurrence is associated with increased risk of chronic kidney disease (CKD), reduced quality of life, and even death [13–16]. Therefore, AKI is a critical public health concern. The incidence of AKI is higher than that of acute lung injury or severe sepsis, and AKI is associated with adverse clinical outcomes and high medical costs, including increased mortality, increased hospitalization periods, and the increased risk of requirement for chronic dialysis in survivors [17–19]. The prevalence of AKI has been increasing in South Korea, and this phenomenon combined with the aging of the population poses an economic and social burden (Fig. 1).

The primary underlying mechanism for pre-renal AKI is a series of impairment in renal autoregulation related to pre-glomerular arteriolar vasodilation, by prostaglandin I2 and nitric oxide and post-glomerular arteriolar vasoconstriction, by angiotensin II [20, 21]. True hypovolemia or a reduction in the effective circulating volume, such as decreased cardiac output, systemic vasodilation, or intra-renal vasoconstriction results in impaired renal autoregulation, leading to decreased glomerular filtration rate, which is proportional to the level of hypoperfusion [22, 23]. Dehydration due to exposure to high temperature may lead to decreased intravascular volume, increased vascular resistance, or low cardiac output, which eventually lead to hemodynamically mediated (pre-renal) AKI [24]. Moreover, acute volume depletion leads to increased proximal reabsorption, which affects tubulo-glomerular feedback, resulting in a pre-renal reduction in glomerular filtration rate [25].

Numerous studies have investigated the association between ambient temperature and morbidity, particularly renal function. Most of these studies have utilized hospital admission data to demonstrate that renal morbidity rates are associated with temperature increases [5, 7, 8, 26, 27]. However, at our location, hospital admissions would not be appropriate for assessing the acute short-term association in a time transient study because most admissions are scheduled in Seoul. Inclusion of scheduled admissions could attenuate observed associations with ambient temperature, due to inclusion of admissions for which timing of the event was not caused by ambient temperature. Therefore, in this research, emergency department (ED) data for unscheduled visits were used as the outcome measure to gain a better understanding of the relationship between ambient temperature and AKI morbidity in Seoul, a city with a temperate climate with distinct seasons. We performed a time-stratified case-crossover analysis based on conditional logistic regression to investigate the association. In addition, we examined the shape of the associations as well as lag effects. To our knowledge, this is the first study to assess the generalizability of the association between ambient temperature and AKI using ED visit data in Seoul.

## Methods

### Study location and health outcomes

This study was conducted in Seoul (37.34°N, 126.59°E), which is the capital and largest metropolis of South Korea with a population of approximately 10 million [28]. Seoul spans a land area of 605.25 km^2^, which is only 0.6% of the total area of South Korea, but 1/5 of the total population of South Korea live in Seoul (16,492 person/km^2^). Seoul has a temperate climate with distinct seasons and a wide range of temperatures across the year.

We utilized data on ED visits recorded by the National Emergency Medical Center (NEMC). The NEMC is a government-funded national ED control agency, and one of its roles is to collect data on ED visits to improve the quality of emergency medical service and health care. The collected data included patient’s information such as sex, age, type of insurance, level of consciousness, vital signs, means of transportation, emergency operative procedures, time variables (visit, discharge, and admission), critical care requirement, disposition status after the ED encounter, duration of hospitalization, and final outcomes (information regarding discharge, transfer, and death) [29]. These data are transferred electronically from the hospitals to the NEMC via a National Emergency Department Information System (NEDIS). The agency maintains an accurate assessment system and annually reports the results to the Ministry of Health and Welfare [29]. The NEDIS database has been widely used by broad range of epidemiological researchers due to its reliability [30]. ED visit data were obtained from the NEDIS of the NEMC between January 1, 2010 and December 31, 2014 for this study. ED visit data were coded and classified according to the discharge diagnosis using the International Classification of Disease 10th Revision (ICD-10). Patients with ICD-10 code N17 based on the primary and secondary disease codes were considered to have AKI (Additional file 1: Table S2). Because the data were based on ED visit incidence, the onset was regarded as acute even in case of CKD, although it could be regarded as an acute exacerbation of CKD. In this sense, we examined acute renal illness as a whole. In addition, we stratified the patients with both CKD (ICD-10 code N18) and AKI (ICD-10 code N17) in the sensitivity analysis due to the possible discrepancies in the pathophysiological mechanisms of AKI with pre-existing CKD (Additional file 1: Figure S1). Patients were also stratified by sex (male and female), age (< 65 years and ≥ 65 years), and season (warm and cool) when they visited the ED. We also analyzed the comorbidities for AKI, namely, hypertension and diabetes.

### Environmental variables

Automated Synoptic Observing System (ASOS) data from 2010 to 2014 were obtained from the Korean Meteorological Administration (KMA). ASOS collects data every minute, including temperatures (°C), relative humidity (%), and air pressure (hPa), and KMA provides city-level daily average of these meteorological variables. Because air pollution has been reported to have a short-term effect on renal morbidity [31], we also obtained hourly concentrations of particulate matter with an aerodynamic diameter of < 10 μm (PM_10_) from 27 monitoring sites operated by the Korean National Institute of Environmental Research. Hourly mean concentrations across the monitoring sites were calculated by averaging monitor-specific concentrations, then we calculated the daily representative concentrations of PM_10_ by averaging the 24-h values from all monitoring stations in Seoul. The data were grouped into two seasons: warm (April–September) and cool (October–March).

### Statistical analysis

We used a time-stratified case-crossover design based on conditional logistic regression to analyze the short-term effects of temperature on AKI-ED visits. The case-crossover design, which is a variant of the case-control design, is largely used in environmental epidemiology research [32] for evaluating when the outcome is acute and the exposure is transient [33]. Comparisons were made between the case day (the day of the case visits ED) and several control days. In this way, each patient serves as his/her own control on days other than the case day with measured and unmeasured potential confounding factors such as age, sex, smoking status, and other genetic predisposition. Moreover, these are automatically controlled by perfect matching. The control days were selected as the same month and year and matched by day of week for each case. This time-stratified method of selecting comparison days avoids bias resulting from time trends in examination of the environmental exposures. Long-term and seasonal time trends and day of the week were also controlled by design [34]. We included potential time-varying confounders, which are relative humidity and barometric pressure in the model. We also performed sensitivity analyses to examine the confounding effects of PM_10_ (lag 0–1).

To explore the susceptibility of groups to the influence of temperature, we modelled the interactions between temperature and each subgroup, namely, age, sex, comorbidities (hypertension and diabetes), and season, to determine whether the effects of temperature differ in these aspects because the case-crossover design cancelled out the potential confounding time-invariant variables.

The exposure–response curve of the relationship between ambient temperature and ED visits was also explored for both warm and cool seasons. For the nonlinear (J-shaped) relationship of the warm season, we fitted value for each observations and the penalized spline curve from conditional logistic model and conducted a piecewise linear regression analysis to estimate the threshold temperature [35]. Using a grid search method with a threshold temperature range of 17 °C–27 °C, we found the point that produces the minimum Akaike information criterion among the equally spaced grid points, 0.1 °C [36]. The lag effect, lagged by up to 7 days before ED visit, was also analyzed because many studies found a short latency of the effect of temperatures on morbidity [37]. The overall associations are estimated as odds ratios (OR) with 95% confidence intervals (CI) per 1 °C increase in temperature. Moreover, we tested for the significant difference between estimates in each subgroup as shown below [38, 39].

$$ {\hat{E}}_1-{\hat{E}}_2\pm 1.96\times \sqrt{{{\hat{SE}}_1}^2+{{\hat{SE}}_2}^2}. $$

A significance level of *α* = 0.05 was adopted for each test. Statistical analysis was conducted using R software version 3.1.0 with the survival package (R Foundation for Statistical Computing, http://www.R-project.org).

## Results

The characteristics of the patients who visited the ED for AKI during the 5-year study period (2010–2014) are shown in Table 1. There were 21,656 cases with 73,755 controls, yielding 3 or 4 control days for each patient. Of these, 12,465 visits (57.56%) were by men, and 13,516 visits (62.41%) were by patients aged 65 years or older. The number of ED visits due to AKI did not vary by season (*X*^2^ = 0.2, *P* = 0.6538). The daily mean temperature was 12.49 °C (standard deviation (SD) ±11.03) for the overall study period, 21.16 °C (SD ± 5.75) for the warm season (April–September), and 3.79 °C (SD ± 7.71) for the cold season (October–March). The daily mean relative humidity was 60.43% for the overall study period and was higher for the warm season (65.01%, SD ± 15.20) than that for the cool season (55.84%, SD ± 13.64). The daily mean concentration of particulate matter (PM) with aerodynamic diameter less than 10 μm (PM_10_) was 47.08 μg/m^3^ (SD ± 27.35) for the overall study period (Table 2). The concentration of PM_10_ in Seoul is relatively high in spring and winter and relatively low in summer and fall. The monthly summary can be found in Additional file 1: Table S1.

**Table 1:** Demographic characteristics of patients with acute kidney injury who visited the emergency department

Characteristics		No. of subjects	(%)	*χ* ^2^	*p*-value
Total		21,656	(100)		
Sex	Male	12,465	(57.56)	494.97	< 0.0001
	Female	9191	(42.44)		
Age (years)	< 65	8140	(37.59)	1334.6	< 0.0001
	≥ 65	13,516	(62.41)		
Season	Warm	10,861	(50.15)	0.2	0.6538
	Cool	10,795	(49.85)		
Comorbidities	AKI with coexisting hypertension	5245	(24.22)		
	AKI with coexisting diabetes	3508	(16.20)

**Table 2:** Descriptive statistics for environmental variables in Seoul, South Korea, 2010–2014

Environmental variables	Mean (SD)		
	Overall	Warm	Cool
Mean temperature (°C)	12.49 (11.03)	21.16 (5.75)	3.79 (7.71)
Mean relative humidity (%)	60.43 (15.15)	65.01 (15.20)	55.84 (13.64)
Mean pressure (hPa)	1005.86 (7.74)	1000.27 (5.40)	1011.48 (5.29)
Mean PM_10_ (μg/m^3^)	47.08 (27.35)	42.28 (25.06)	51.91 (28.68)

**Fig. 2:**
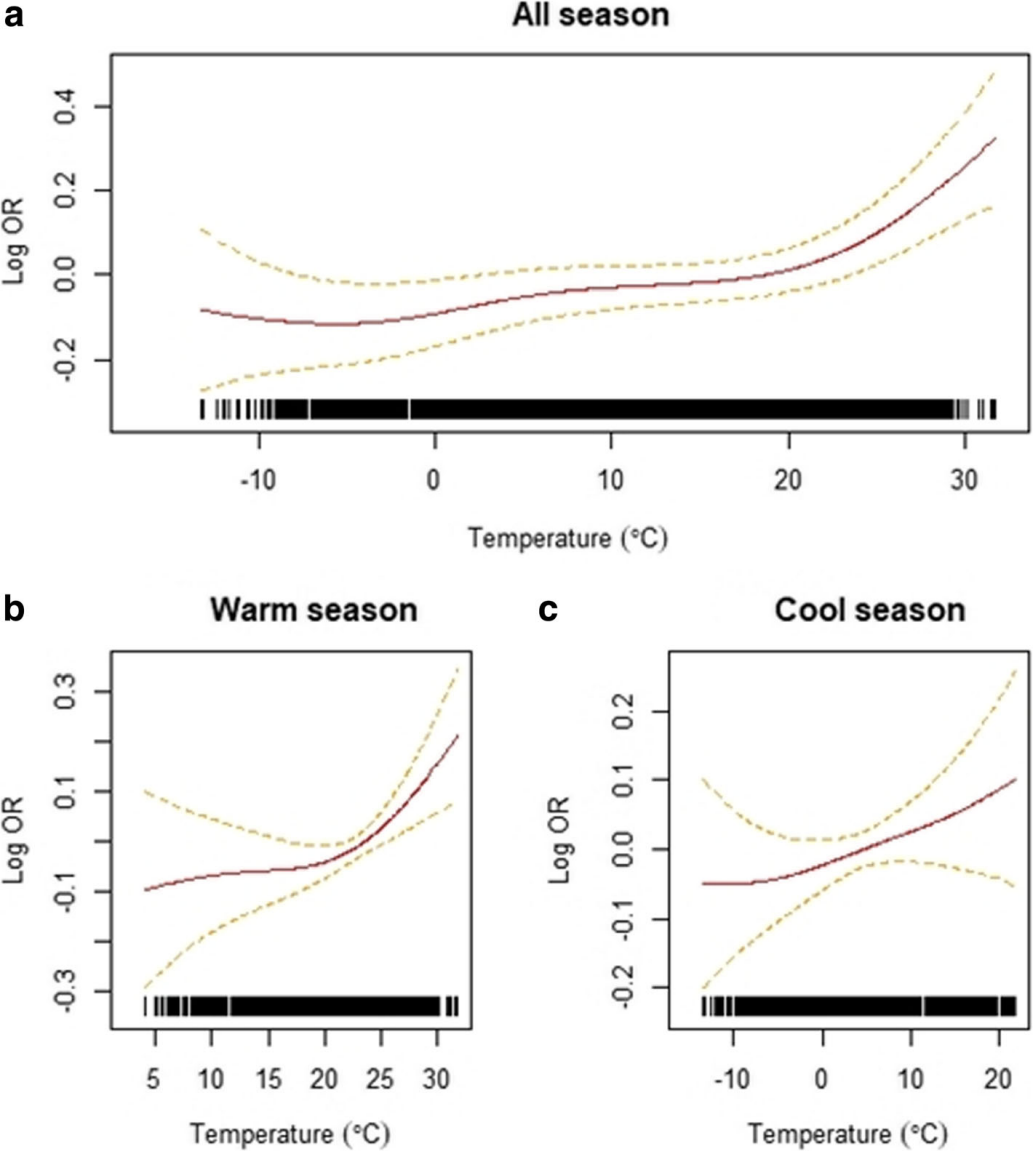
Relationship between ambient temperature and risks of emergency department visits due to acute kidney injury in Seoul, South Korea, between 2010 and 2014 in (**a**) all seasons, (**b**) the warm season (April–September), and (**c**) the cool season (October–March). The dotted lines indicate 95% confidence intervals (CIs)

The exposure–response curves between ambient temperature and AKI-ED visits are shown in Fig. 2. Overall, the risk of AKI increases as temperature increases. In particular, the risk sharply increased during the warm season. The threshold temperature was found to be at 22.3 °C from grid searching (Additional file 1: Figure S2), with the OR increasing above this threshold. The OR above 22.3 °C during the warm season was 1.0235 (95% confidence interval [CI]: 1.0230, 1.0239), while the OR below 22.3 °C was 1.0019 (95% CI: 1.0017, 1.0020).

**Fig. 3:**
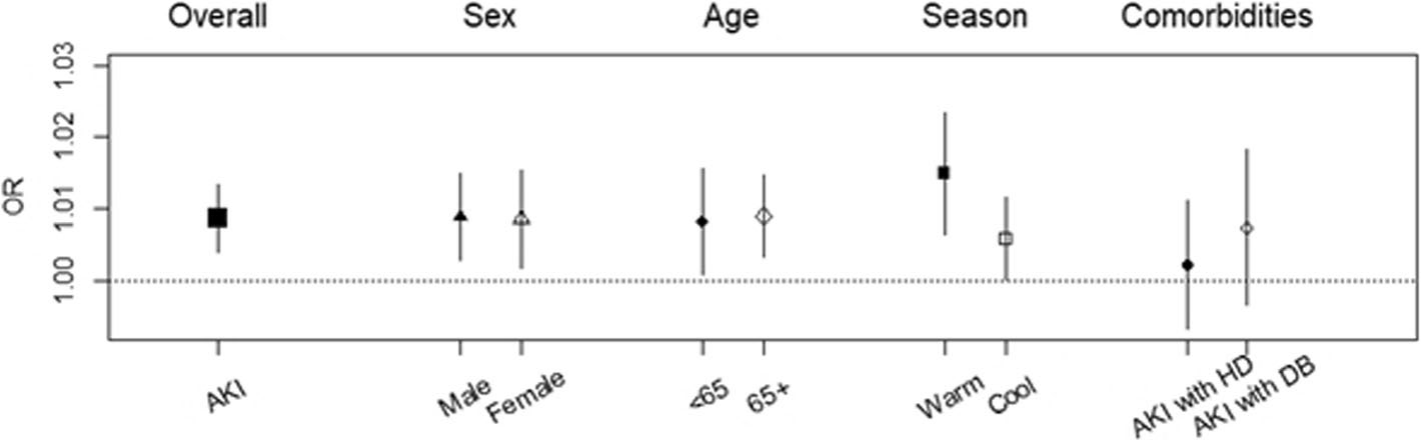
Effects of temperature on emergency department visits for acute kidney injury by subgroups in Seoul, South Korea, from 2010 to 2014. The overall associations are estimated as odds ratios (ORs) with 95% confidence intervals per 1 °C increase in temperature. Adjusted for relative humidity, air pressure, and PM_10_ in the model. Abbreviations: AKI, acute kidney injury; < 65, < 65 years of age; 65+, ≥ 65 years of age; HD, hypertension disease; DB, diabetes

Figure 3 shows the overall, sex-, age-, comorbidity-, and season-specific associations between ambient temperature and AKI from the final model (Additional file 1: Table S2). We illustrated the OR per 1 °C increase in ambient temperature for various categories on a single day (lag 0) because the greatest risk was observed for lag 0 (Additional file 1: Figure S4). There was strong evidence of associations between temperature and AKI (OR = 1.0087, *P <* 0.001). The associations were significant in both sexes (Fig. 3). However, although the risk estimate is higher among men (OR = 1.0088, *P* = 0.004) than among women (OR = 1.0086, *P* = 0.014), the difference between the two groups was not significant (*P* = 0.959). There were also significant associations in both age groups. However, although the association was stronger in those aged ≥ 65 years (OR = 1.0090, *P* = 0.002) than in those aged < 65 years (OR = 1.0083, *P* = 0.027), the difference was not significant (*P* = 0.875). By contrast, there was little evidence of association with comorbidities. The associations were still positive but not significant for both comorbid hypertension (OR = 1.0022, *P* = 0.633) and comorbid diabetes (OR = 1.0074, *P* = 0.181). The associations were significant in both seasons, and the risk estimate was higher during the warm season (OR = 1.0149, *P* = 0.001) than the cool season (OR = 1.0059, *P* = 0.039). In the sensitivity analysis, the effect of ambient temperature was examined with and without PM_10_ in the model, and the temperature effect was robust to the adjustment of PM_10_ (Additional file 1: Figure S3).

**Fig. 4:**
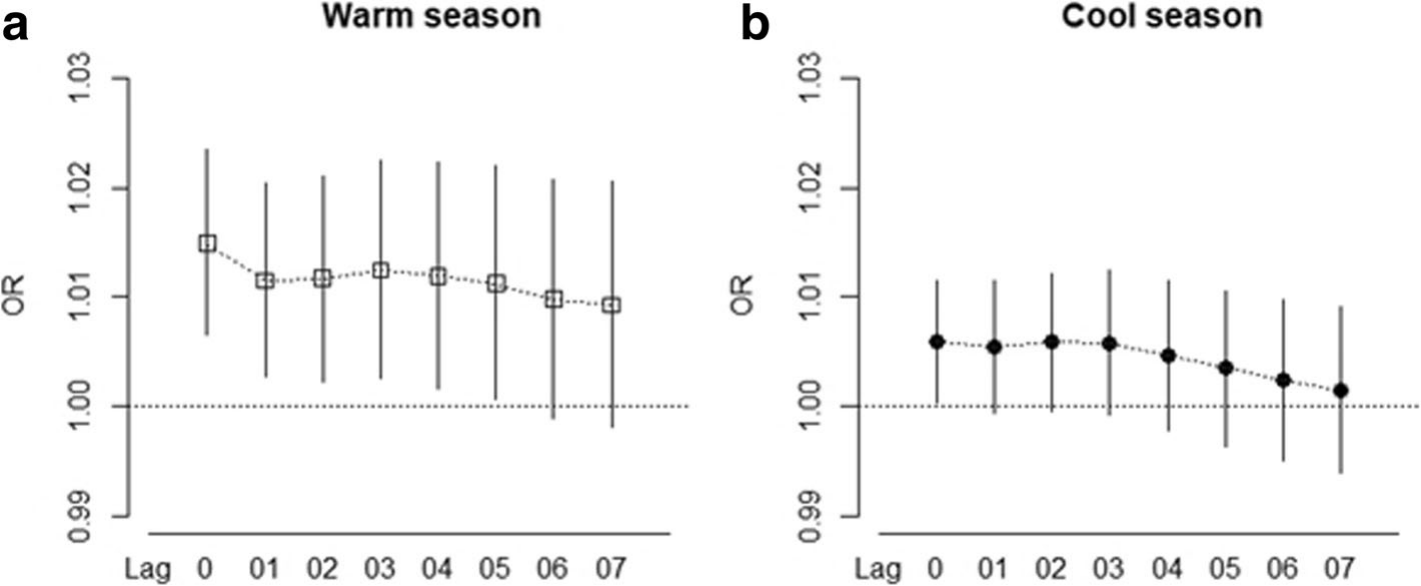
Lag structures by season. The effects of ambient temperatures (°C) on acute kidney injury along lag days during the (**a**) warm season and (**b**) cool season

Figure 4 illustrates the lag patterns for the effect of temperature on AKI-ED visits during the warm and cool season. In both seasons, the temperature effect appeared to be acute and immediate and persisted for a few days. Thus, all subsequent results shown are for lag 0. Similar lag patterns were also observed among subgroups by sex and age (Additional file 1: Figure S4). Within each group, a noticeable delay is also seen for women and patients aged < 65 years at lag 03 with an acute effect at lag 0.

## Discussion

We observed a considerable increase in the risk of AKI that was associated with escalations in ambient temperature over all seasons, and the risk for AKI was particularly higher during the warm season. In addition, a noticeable nonlinear relationship with temperature was found during the warm season, indicating a severe risk for AKI associated with ambient temperatures above the threshold of 22.3 °C. Our findings are in line with those of previous studies that show evidence supporting a positive association between high temperatures and the risk of AKI. Two studies from the US and one from Australia found that hospital admissions for AKI were substantially increased during heat wave periods compared with non-heat wave periods [8, 27, 40]. In addition, a study of temperature effects on AKI hospital admissions in California reported a 7.4% increase in AKI admissions associated with each 10 °F (5.56 °C) increase in daily mean temperature [7], while 8.28% increase in AKI-ED visits associated with each 10 °F during the warm season in Seoul of our study. Given that our results were obtained using individual ED visit data, this study strengthens the epidemiologic evidence of an acute adverse effect of ambient temperature on AKI morbidity.

We observed considerable adverse effects of temperature on ED visits due to AKI in both warm and cool seasons as the temperature increases, but the risk was greater during the warm season than the cool season and was even greater above the threshold temperature of 22.3 °C. Many studies have focused on the health effects of heatwaves or high temperatures [8, 27, 40, 41]. However, the risk for AKI substantially increased in temperatures above 22.3 °C in the present study, suggesting that the threshold temperature is lower than that used in previous studies. This finding is consistent with that of Kovats et al. [42] who reported that threshold temperatures of 18 °C for diseases of the renal system and 21 °C for renal failure. Additionally, they reported that kidney stones have a considerable effect on hospital admissions for renal disease in Greater London, UK. This suggests that not only extremely hot temperatures and heatwaves, but also moderate temperatures affect health. The effects of non-extreme weather are less focused in the literature. The increased risk of AKI from outdoor exposures during the warm season places greater emphasis on the preventive aspects of AKI.

Some studies explored the temporal lag patterns of the association between temperature and health risks. For the association of temperature with morbidity, lag days were reported ranging from the same day [3] to a month [43]. According to Fletcher et al. (2012), the strongest association between the mean temperature and AKI hospital admissions occurs at lag 1 (OR = 1.09, 95% CI: 1.07, 1.12), while significant associations were also observed at lags 0 and 2 (OR = 1.06, 95% CI: 1.04, 1.09 and OR = 1.06, 95% CI: 1.03, 1.08, respectively). Our result showed more acute effects of temperature on AKI-ED visits for lag 0 in both seasons. Similarly, Basu et al. found acute effects of temperature (lag 0) on ED visits in California, USA [3]. In Atlanta, USA, Chen et al. also found that increased temperature had same-day (lag 0) effects on both all renal diseases and AKI [44].

PM_10_ was assessed in the model. Associations between PM and daily mortality [45], cardiovascular hospital admissions in the elderly [46], and infant mortality [47] have been identified in Korean cities. Furthermore, a recent longitudinal study among US veterans demonstrated the associations of PM concentrations with a higher risk of reducing renal function [31], development of CKD, and progression to end-stage renal disease [48]. It is biologically plausible that the inflammation and oxidative stress linked to PM exposure could be an underlying mechanism for a broader number of disease outcomes [49], including renal disorders. Nonetheless, the effect of temperature on AKI was robust with a potential confounder of air pollutants in our analysis. Moreover, our findings remained statistically significant after adjusting for PM_10_. In addition, the fact that the temperature effect is robust to the adjustment for PM in our analysis suggests that PM is not solely responsible for the higher risk of AKI. Toxicity depends on the components of PM [50], and exposure patterns vary by seasons due to behavioral changes of individuals [51]. Thus, the adverse effects of PM on AKI need to be further validated.

Contrary to expectations, the temperature effects of AKI did not vary substantially across demographic characteristics. The elderly may be more vulnerable due to a reduction in thermoregulatory abilities, age-related declines in kidney function, adaptation behaviors, low self-care abilities, and health comorbidities. However, although the risk of the elderly was slightly higher among those aged ≥ 65 years than among those aged < 65 years, the difference was not statistically significant (*P* = 0.87). No significant difference by sex was noted in this study either (*P* = 0.96).

On the other hand, we also examined the susceptibility to ambient temperatures of persons with comorbidities of prevalent chronic illnesses, particularly hypertension and diabetes. Although AKI is more prevalent among individuals who already have hypertension or diabetes [52], our analysis of temperature-related AKI-ED visits was not significantly associated with these comorbidities. Further in-depth studies are required to clarify these discrepancies.

Even small acute changes in kidney function can result in both short- and long-term complications. Early diagnosis and appropriate treatment of AKI are associated with an increased survival rate and restore complete renal function. This results in reduced cost of treatment [53] because CKD patients require hemo- or peritoneal dialysis or kidney transplantation. If AKI patients without premorbid CKD survive, then most of them recover to dialysis independence [54]. Therefore, early diagnosis and appropriate treatment is crucial to prevent subsequent CKD, end-stage renal disease, or death [55] in AKI patients.

This study furthers our understanding of the association between temperature and AKI in Seoul. However, our study has some limitations, including the possible misclassification of exposures intrinsic in ecological studies. The use of ambient rather than personal measurements of temperature may have resulted in bias, which probably underestimated the association. Also, differences between indoor and outdoor temperatures due to air conditioning or heating may affect the association between temperature and AKI outcomes. Similar to other environmental epidemiological studies on AKI [26], we used the ICD-10 code for the definition of AKI without knowing whether the standard Kidney Disease Improving Global Outcomes (KDIGO) criteria were used for diagnosis. With ICD-codes, diagnosis of AKD could be incorrectly reported or underreported [56]. We did not have information on medication, as nephrotoxic medications contribute to a substantial proportion of AKI. Further, there may be other individual factors influencing the association between air temperature and AKI. Finally, our study was conducted in a single city; therefore, the findings may not be applicable to other target populations in other areas. To explicitly understand the effects of temperature on AKI, multi-city studies are required.

Undoubtedly, AKI is affected by more complex factors aside from ambient temperature. However, despite these limitations, we found convincing evidence supporting that temperature might be a triggering or exacerbating factor for AKI. The findings from this study have considerable public health implications because it may help elucidate the effects of ambient temperature on AKI.

## Conclusions

Based on the estimation of the impact of temperature on ED visits of patients with AKI in Seoul, a considerable health burden at elevated temperatures was identified for this population. Projections from global climate models indicate that the variability and extremes of temperature that may affect AKI are likely to increase in the future. Thus, the relationship between temperature and AKI needs to be investigated. Our findings suggest that increases in temperatures are a risk factor for AKI. Patient management and education need to be improved as extreme temperatures become more prevalent with climate change.

## Additional file

Additional file 1: **Table S1.** Descriptive statistics for environmental variables by months between 2010 and 2014. **Table S2.** Effects of temperature on emergency department visits for acute kidney injury showing odds ratios, 95% confidence intervals, and *p*-values. **Figure S1.** Effects of temperature on emergency department visits for acute kidney injury including and excluding the patient with chronic kidney disease. The overall associations are estimated as odds ratios (OR) with 95% confidence intervals per 1°C increase in temperature. Considerable differences are not observed. Abbreviations: AKI, acute kidney injury; CKD, Chronic Kidney Disease. **Figure S2.** Threshold point estimation (22.3°C) using grid search methods during the warm season. The approximate range of the threshold temperature was between 19°C and 27°C with grid points 0.1°C. (a) Fitted value from conditional logistic model. (b) Penalized spline curve for temperature term in the conditional logistic model. (c) Predicted curve for temperature term. (d) Piecewise linear regression that minimizes the Akaike information criterion value with *β*_*1*_ = 0.0019 (odds ratio [OR] = 1.0019, 95% confidence interval [CI]: 1.0017, 1.0020), *β*_*2*_ = 0.0232 (OR = 1.0235, 95% CI: 1.0230, 1.0239). Abbreviation: AIC, Akaike information criterion. **Figure S3.** Effects of temperature on emergency department visits for acute kidney injury with and without adjustment of PM10 in the model by subgroups in Seoul, South Korea, between 2010–2014. The overall associations are estimated as odds ratios (OR) with 95% confidence intervals per 1°C increase in temperature. Adjusted for relative humidity and air pressure in the model. Abbreviations: AKI, acute kidney injury; < 65, < 65 years of age; 65+, ≥ 65 years of age; HD, hypertension disease; DB, diabetes. **Figure S4.** Lag structure by subgroups. The effects of ambient temperatures (°C) on acute kidney injury along days of lag in (a) men, (b) women, (c) age under 65 years, and (d) age above 65 years. (DOCX 109 kb)

## Data Availability

ED data that support the findings of this study are available from NEMC but restrictions apply to the availability of these data, which were used under license for the current study, and so are not publicly available. Data are however available from the authors upon reasonable request and with permission of NEMC.

## Abbreviations

AKI: Acute Kidney Injury
ASOS: Automated Synoptic Observing System
CI: Confidence Interval
CKD: Chronic Kidney Disease
ED: Emergency Department
ICD: International Classification of Disease
IRB: Institutional Review Board
KDIGO: Kidney Disease Improving Global Outcomes
KMA: Korean Meteorological Administration
NEDIS: National Emergency Department Information System
NEMC: National Emergency Medical Center
OECD: Organization for Economic Co-operation and Development
OR: Odds Ratio
PM: Particulate Matter
SD: Standard Deviation
