# Supplementary material for: Temperature as a risk factor of emergency department visits for acute kidney injury: a case-crossover study in Seoul, South Korea
Source: Environ Health. 2019 Jun 14;18:55. doi: 10.1186/s12940-019-0491-5 (PMC6570878; doi:10.1186/s12940-019-0491-5)
Supplement: Supplementary file 1 — Table S1. Descriptive statistics for environmental variables by months between 2010 and 2014. Table S2. Effects of temperature on emergency department visits for acute kidney injury showing odds ratios, 95% confidence intervals, and p-values. Figure S1. Effects of temperature on emergency department visits for acute kidney injury including and excluding the patient with chronic kidney disease. The overall associations are estimated as odds ratios (OR) with 95% confidence intervals per 1°C increase in temperature. Considerable differences are not observed. Abbreviations: AKI, acute kidney injury; CKD, Chronic Kidney Disease. Figure S2. Threshold point estimation (22.3°C) using grid search methods during the warm season. The approximate range of the threshold temperature was between 19°C and 27°C with grid points 0.1°C. (a) Fitted value from conditional logistic model. (b) Penalized spline curve for temperature term in the conditional logistic model. (c) Predicted curve for temperature term. (d) Piecewise linear regression that minimizes the Akaike information criterion value with β1 = 0.0019 (odds ratio [OR] = 1.0019, 95% confidence interval [CI]: 1.0017, 1.0020), β2 = 0.0232 (OR = 1.0235, 95% CI: 1.0230, 1.0239). Abbreviation: AIC, Akaike information criterion. Figure S3. Effects of temperature on emergency department visits for acute kidney injury with and without adjustment of PM10 in the model by subgroups in Seoul, South Korea, between 2010–2014. The overall associations are estimated as odds ratios (OR) with 95% confidence intervals per 1°C increase in temperature. Adjusted for relative humidity and air pressure in the model. Abbreviations: AKI, acute kidney injury; < 65, < 65 years of age; 65+, ≥ 65 years of age; HD, hypertension disease; DB, diabetes. Figure S4. Lag structure by subgroups. The effects of ambient temperatures (°C) on acute kidney injury along days of lag in (a) men, (b) women, (c) age under 65 years, and (d) age above 65 years. (DOCX 109 kb) [file 12940_2019_491_MOESM1_ESM.docx]

Additional file 1

**Temperature as a risk factor of emergency department visits for acute kidney injury: A case-crossover study in Seoul, South Korea.**

**Contents**

Table S1. Descriptive statistics for environmental variables by months between 2010 and 2014

Table S2. Effects of temperature on emergency department visits for acute kidney injury showing odds ratios, 95% confidence intervals, and *p*-values

Figure S1. Effects of temperature on emergency department visits for acute kidney injury including and excluding the patient with chronic kidney disease. The overall associations are estimated as odds ratios (OR) with 95% confidence intervals per 1°C increase in temperature. Considerable differences are not observed. Abbreviations: AKI, acute kidney injury; CKD, Chronic Kidney Disease

**Figure S2.** Threshold point estimation (22.3°C) using grid search methods during the warm season. The approximate range of the threshold temperature was between 19°C and 27°C with grid points 0.1°C. (**a**) Fitted value from conditional logistic model. (**b**) Penalized spline curve for temperature term in the conditional logistic model. (**c**) Predicted curve for temperature term. (**d**) Piecewise linear regression that minimizes the Akaike information criterion value with *β_1_* = 0.0019 (odds ratio [OR] = 1.0019, 95% confidence interval [CI]: 1.0017, 1.0020), *β₂* = 0.0232 (OR = 1.0235, 95% CI: 1.0230, 1.0239). Abbreviation: AIC, Akaike information criterion

Figure S3. Effects of temperature on emergency department visits for acute kidney injury with and without adjustment of PM_10_ in the model by subgroups in Seoul, South Korea, between 2010–2014. The overall associations are estimated as odds ratios (OR) with 95% confidence intervals per 1°C increase in temperature. Adjusted for relative humidity and air pressure in the model. Abbreviations: AKI, acute kidney injury; < 65, < 65 years of age; 65+, ≥ 65 years of age; HD, hypertension disease; DB, diabetes

Figure S4. Lag structure by subgroups. The effects of ambient temperatures (°C) on acute kidney injury along days of lag in (a) men, (b) women, (c) age under 65 years, and (d) age above 65 years

Table S1. Descriptive statistics for environmental variables by months between 2010 and 2014

| Month | Mean (SD) | | | | | |
| --- | --- | --- | --- | --- | --- | --- |
|  | **Mean temperature (°C)** | **Mean relative humidity (%)** | **Mean pressure (hPa)** | **Total rain (mm)** | **Total sunshine (hr)** | **Mean PM_10_ (μg/m^3^)** |
| Jan | -3.72 (4.41) | 54.90 (13.25) | 1014.89 (4.46) | 1.54 (3.23) | 5.99 (3.29) | 58.18 (25.56) |
| Feb | 0.24 (4.80) | 52.44 (15.06) | 1012.74 (4.77) | 4.28 (9.33) | 6.27 (3.43) | 57.04 (30.99) |
| Mar | 5.20 (3.79) | 54.13 (13.72) | 1009.11 (5.24) | 3.20 (4.86) | 6.68 (3.93) | 59.26 (35.73) |
| Apr | 11.31 (3.70) | 55.25 (15.8) | 1004.67 (5.47) | 7.87 (12.82) | 6.72 (4.40) | 55.13 (23.59) |
| May | 18.40 (3.05) | 56.73 (14.59) | 1000.86 (5.09) | 6.68 (12.03) | 7.43 (4.57) | 61.04 (35.12) |
| Jun | 23.40 (2.13) | 63.06 (14.15) | 997.57 (3.85) | 11.91 (26.47) | 6.76 (4.20) | 44.31 (19.91) |
| Jul | 25.50 (1.80) | 75.91 (9.32) | 996.42 (4.32) | 26.49 (44.55) | 3.79 (3.94) | 33.77 (17.47) |
| Aug | 26.45 (2.19) | 73.31 (10.87) | 998.62 (3.86) | 15.83 (26.64) | 4.63 (4.15) | 30.47 (14.67) |
| Sep | 21.67 (2.72) | 65.41 (13.01) | 1003.63 (4.00) | 20.64 (40.30) | 6.15 (4.27) | 29.01 (12.41) |
| Oct | 15.10 (3.27) | 59.03 (12.7) | 1009.30 (4.31) | 7.18 (11.20) | 7.37 (3.38) | 37.27 (14.05) |
| Nov | 7.56 (4.61) | 58.34 (13.76) | 1010.46 (4.70) | 3.28 (5.79) | 5.82 (3.51) | 48.99 (29.07) |
| Dec | -1.87 (4.51) | 55.96 (12.42) | 1012.44 (5.63) | 1.65 (3.26) | 5.98 (3.27) | 51.10 (26.47) |

Table S2. Effects of temperature on emergency department visits for acute kidney injury showing odds ratios, 95% confidence intervals, and *p*-values

| Indication |  | ICD 10 code | OR (95% CI) |
| --- | --- | --- | --- |
| AKI |  | N17 | 1.0087 (1.0041, 1.0134)* |
| Sex | Male |  | 1.0088 (1.0029, 1.0148)* |
|  | Female |  | 1.0086 (1.0018, 1.0155)* |
| Age | < 65 |  | 1.0083 (1.0009, 1.0156)* |
|  | ≥ 65 |  | 1.0090 (1.0033, 1.0148)* |
| Season | Warm |  | 1.0149 (1.0065, 1.0234)* |
|  | Cool |  | 1.0059 (1.0003, 1.0116)* |
| Comorbidities | AKI with coexisting hypertension | N17 & I10-I15 | 1.0022 (0.9933, 1.0112) |
|  | AKI with coexisting diabetes | N17 & E10-E14 | 1.0074 (0.9966, 1.0184) |

OR, odds ratio; CI, confidence interval; AKI, acute kidney injury

* *p*-value < 0.05


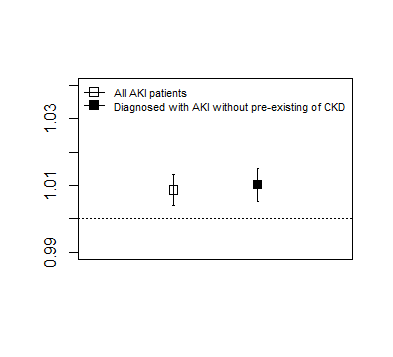


Figure S1. Effects of temperature on emergency department visits for acute kidney injury including and excluding the patient with chronic kidney disease. The overall associations are estimated as odds ratios (OR) with 95% confidence intervals per 1°C increase in temperature. Considerable differences are not observed. Abbreviations: AKI, acute kidney injury; CKD, Chronic Kidney Disease


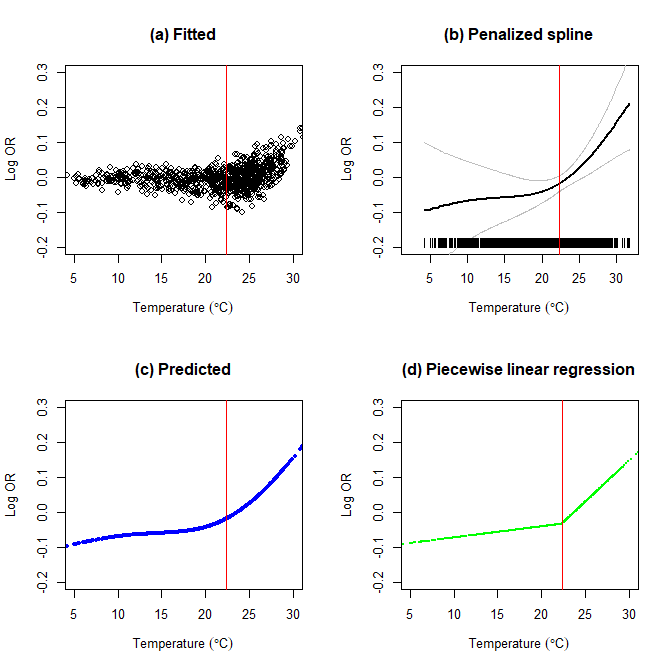


**Figure S2.** Threshold point estimation (22.3°C) using grid search methods during the warm season. The approximate range of the threshold temperature was between 19°C and 27°C with grid points 0.1°C. (**a**) Fitted value from conditional logistic model. (**b**) Penalized spline curve for temperature term in the conditional logistic model. (**c**) Predicted curve for temperature term. (**d**) Piecewise linear regression that minimizes the Akaike information criterion value with *β_1_* = 0.0019 (odds ratio [OR] = 1.0019, 95% confidence interval [CI]: 1.0017, 1.0020), *β₂* = 0.0232 (OR = 1.0235, 95% CI: 1.0230, 1.0239). Abbreviation: AIC, Akaike information criterion


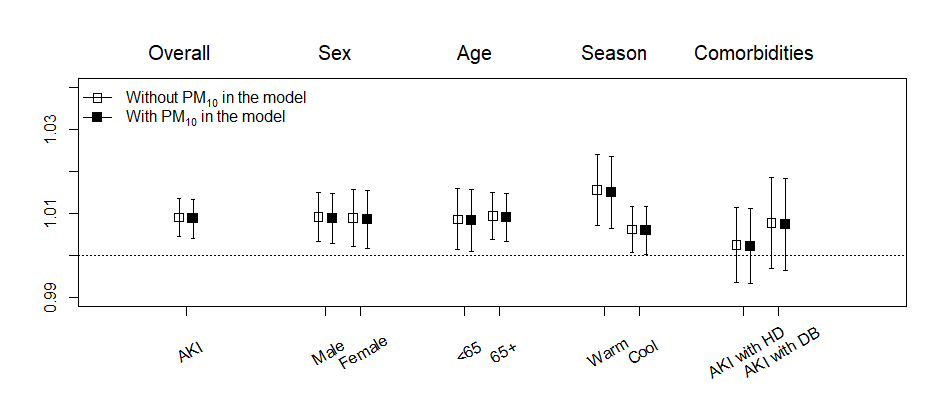


Figure S3. Effects of temperature on emergency department visits for acute kidney injury with and without adjustment of PM_10_ in the model by subgroups in Seoul, South Korea, between 2010–2014. The overall associations are estimated as odds ratios (OR) with 95% confidence intervals per 1°C increase in temperature. Adjusted for relative humidity and air pressure in the model. Abbreviations: AKI, acute kidney injury; < 65, < 65 years of age; 65+, ≥ 65 years of age; HD, hypertension disease; DB, diabetes


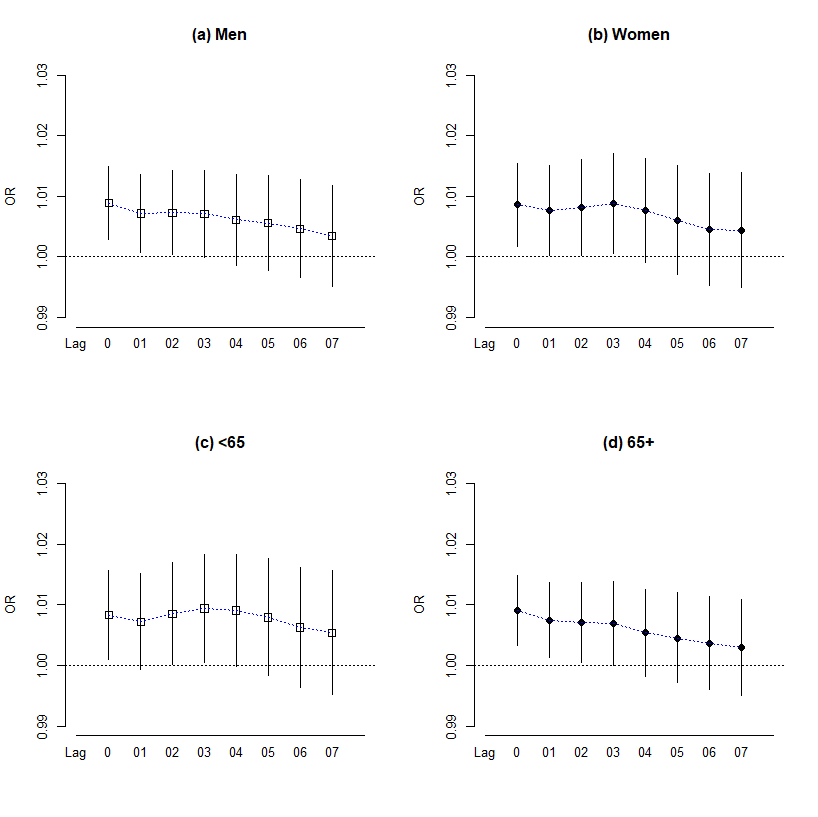


Figure S4. Lag structure by subgroups. The effects of ambient temperatures (°C) on acute kidney injury along days of lag in (a) men, (b) women, (c) age under 65 years, and (d) age above 65 years
